# Dynamic analysis of composition, insecticidal, and antifungal activities of *Zanthoxylum armatum* DC. at different harvesting periods

**DOI:** 10.3389/fpls.2025.1603963

**Published:** 2025-07-18

**Authors:** Yaqin Peng, Danping Xu, Wenkai Liao, Qianqian Qian, Junhao Wu, Tingjiang Gan, Zhihang Zhuo

**Affiliations:** ^1^ College of Life Science, China West Normal University, Nanchong, China; ^2^ Engineering Research Center of Chuanxibei Rural Human Settlement Construction, Mianyang Teachers’ College, Mianyang, China

**Keywords:** *Z. armatum* DC., GC-MS, essential oil, *T. castaneum*, *C. cassiicola*

## Abstract

**Introduction:**

Essential oils from plants contain various volatile compounds with antifungal and antioxidant properties. The synthesis and accumulation of these volatile compounds are closely related to factors such as the plant's geographical origin and harvest period. Investigating the insect-repellent and antimicrobial effects of *Zanthoxylum armatum* DC. essential oils (EOs) at different harvest stages can optimize its harvest and utilization while also promoting the development of eco-friendly agents.

**Methods:**

This study analyzed the changes in the composition and content of volatile compounds in *Z. armatum* EOs at different growth stages in Nanchong City using GC-MS.

**Results:**

The results indicate that the accumulation period of volatile compounds occurs before the t5 stage (August 4). Linalool, D-Limonene, and Sabinene were the three most abundant volatile components in the essential oil of *Z. armatum* pericarp. Many monoterpenes, such as α-Pinene, Sabinene, and β-Myrcene, were found in higher concentrations during the early stages of fruit maturation. Principal component analysis (PCA) revealed a significant difference in volatile composition between the t3, t4, and t5 (t3: July 3, t4: July 18, t5: August 4) stages and the t1, t2, t6, (t1: May 26, t2: June 16, t6: September 9) and t7 (September 28) stages of *Z. armatum*. Volatile compounds were relatively higher in samples collected in July and August, making these months the optimal harvest period for processing and manufacturing related products. As the fruit of *Z. armatum* matures, the content of structurally more complex compounds, such as alcohols and esters, increases. The insect-repellent and antifungal experiments demonstrated that *Z. armatum* EOs exhibited a strong repellent effect against *T. castaneum*, although the EO’s toxicity was not lethal. The antifungal effect was most pronounced in the EO collected during the t4 stage, where the relative content of various antifungal compounds was higher.

**Discussion:**

This suggests that the antifungal activity of the EOs may result from synergistic or antagonistic interactions among its compounds. By exploring the composition, content, and bioactivities (insect-repellent and antifungal) of *Z. armatum* EOs at different harvest periods, this study provides theoretical support for developing market-oriented commercial products.

## 1 Introduction

Essential oils (EOs) are typically extracted from various parts of plant materials, such as flowers, buds, seeds, leaves, branches, bark, herbs, wood, fruits, and roots. They are aromatic, oil-like liquids that are volatile in nature (Burt, 2004). EOs contain a variety of low molecular weight volatile components, such as terpenes and terpenoid compounds, phenolic-derived aromatic components, and fatty components (Bakkali et al., 2008). At different maturity stages, the components of EOs change with the plant’s physiological metabolism. Research by Wu et al. demonstrated that the contents of 3-carene, α-pinene, and β-pinene in *Citrus medic*a EO vary significantly across different stages (Zhen et al., 2013). EOs contain a variety of bioactive plant secondary metabolites that can inhibit or slow the growth of harmful microorganisms (Shenglan et al., 2022). They play a crucial role in plant defense against pathogens and insects. Plant essential oils, as a natural product, are commonly used as natural antimicrobial agents. The essential oils of certain aromatic plants are widely used as food preservatives, helping to extend shelf life and improve the quality of stored foods (Gutierrez et al., 2009). Bhanu et al (Prakash et al., 2012). investigated the antifungal, anti-aflatoxin, and antioxidant properties of *Zanthoxylum alatum* Roxb. essential oil. They found that the main component of the essential oil, methyl cinnamate, exhibited antifungal and anti-aflatoxin effects at low concentrations (0.6 μl/ml), with fungicidal toxicity. Additionally, the essential oil showed strong antioxidant activity.

*Zanthoxylum armatum* DC. is a common shrub or small tree in the Rutaceae family, with approximately 250 species of Zanthoxylum plants found worldwide. These aromatic plants are native to subtropical and temperate regions globally and are particularly cultivated extensively in China, Korea, and Japan (Di et al., 2021). Its fruit is green in color and features slightly raised oil gland dots. Due to its enticing aromatic properties, the fruits and extracts of *Z. armatum* are among the key flavoring agents in the food industry (Hong et al., 2017; Shun et al., 2019). Additionally, as a traditional medicinal plant, *Z. armatum* is commonly used to treat wounds, toothaches, stomachaches, nausea, diarrhea, and ascariasis. Studies have indicated that Zanthoxylum plants contain various bioactive compounds, such as essential oils, alkaloids, lignans, coumarins, flavonoids, amides, polyphenols, and sterols. In the pericarp of *Z. armatum*, EOs are abundant and exhibit antioxidant, antibacterial, and anti-inflammatory effects (Gao and Hanbin, 2014). Currently, there is extensive research on the antimicrobial and insecticidal properties of *Z. armatum* EO, but studies focusing on its specific application in agricultural production are relatively scarce. This may be partly due to the inherent lag in practical agricultural activities and partly due to cost-related challenges associated with the research and application of such green agents.

*Tribolium castaneum* (Coleoptera: Tenebrionidae) (Campbell et al., 2022) is a significant cosmopolitan storage pest with a wide range of hosts. Due to its high reproductive rate and long reproductive lifespan, it exhibits one of the highest population growth rates among all stored-product beetles. Consequently, it is distributed in nearly 156 countries worldwide (Campbell et al., 2022). The eggs of this pest are scattered on the surface of grain kernels, within grain crevices, or among debris and fine particles. The eggs are often coated with a sticky substance, causing powder and debris to adhere to them, making them difficult to detect. This insect secretes odorous fluids from its scent glands, imparting a musty odor to its host. Furthermore, its secretions contain the carcinogenic compound benzoquinone (J. D. and Roth, 1953). In the search for pest control agents with limited adverse effects on the environment and non-target organisms, researchers have continually explored plant resources in the hope of discovering new plant-based insecticides with high insecticidal activity to address pesticide challenges. The EOs of *Cymbopogon* spp., *Ocimum* spp., and *Eucalyptus* spp. show great promise as repellents against insects and arthropods (Ravi Kant and Gayatri, 2007). *Corynespora cassiicola* is one of the pathogens causing target spot disease in mulberry trees. This fungal disease significantly impacts the yield and quality of mulberry trees, posing serious threats to the economy and ecological environment (Xinqi, 2023). While chemical control is relatively effective against this pathogen, it still presents certain challenges. Consequently, the development of environmentally friendly agents is expected to become a key focus of future research.

The composition and content of essential oils are influenced by various factors, including the variety, origin, harvest time, and storage year (Qianqian et al., 2024). Therefore, the composition and content of EOs extracted from *Z. armatum* in different regions may vary, and their inhibitory effects on different pests and fungi may also differ. In this study, samples collected from Nanchong City, Sichuan Province, were analyzed using GC-MS to determine the composition and content of volatile oils, and the insect repellent and antimicrobial activities of the essential oil were subsequently validated. The insecticidal and antimicrobial activities of the EOs were then investigated. This study provides the first systematic time-course analysis of volatile compound profiles in *Z. armatum* EOs across seven distinct growth stages (from May to September), revealing previously unreported temporal accumulation patterns. By integrating GC-MS metabolomics, PCA-based chemometrics, and bioactivity assays, we developed an innovative framework that correlates harvest timing with optimal functional properties, thereby overcoming the limitations of traditional compositional studies. This research will provide data support for the development of natural plant-based insecticidal/antimicrobial agents.

## 2 Materials and methods

### 2.1 Plant materials

**Figure 1:**
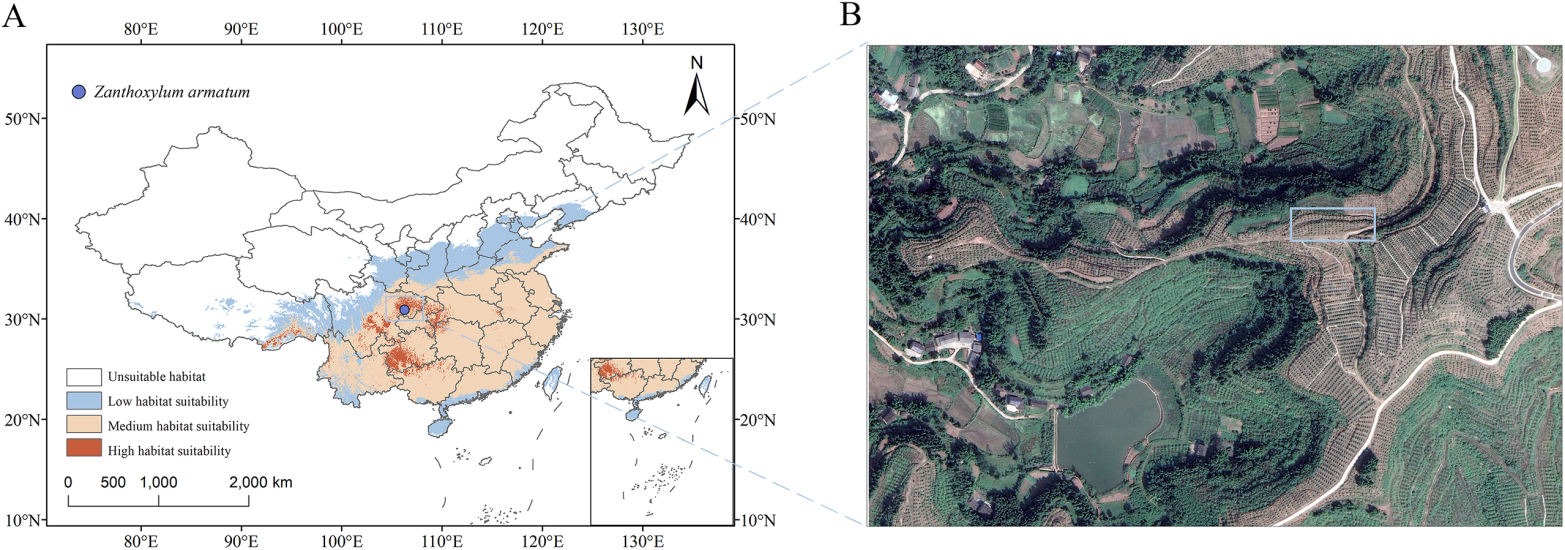
Geographic location map of the sampling points.

The sample collection site is located in the *Z. armatum* plantation in Ca’er Town, Gaoping District, Nanchong City, Sichuan Province (N 30.91438, E 106.28455, Altitude 426 m) (**Figure 1**). *Z. armatum* samples were collected through local forestry bureaus or zanthoxylum planting companies. In the plantation, 20 healthy *Z. armatum* trees aged 2–3 years were randomly selected, and fruit was collected from each tree seven times between May and September. The collection dates were as follows: (t1: May 26, t2: June 16, t3: July 3, t4: July 18, t5: August 4, t6: September 9, t7: September 28). The fresh *Z. armatum* fruit collected during each sampling was dehydrated in an oven at 60 °C for 48 hours. After dehydration, the seeds and pericarps of the *Z. armatum* were separated, placed into sealed bags, and stored at -20 °C for later use. All the specimens were authenticated by Professor Danping Xu of China West Normal University and stored in the School of Life Sciences (NC 20230526), China West Normal University.

### 2.2 Biological materials

*T. castaneum* samples were sourced from the Forest Protection Research Group at the School of Life Sciences, China West Normal University. Healthy adult beetles, 4–7 days post-emergence, were selected for the experiment. The *C. cassiicola* strain, which is responsible for the target spot disease, was obtained from the Sichuan Academy of Agricultural Sciences. The mycelium was inoculated onto PDA agar medium with an inoculation needle and incubated at a constant temperature in an incubator for 3 days before use. All the specimens were authenticated by Professor Zhihang Zhuo of China West Normal University.

### 2.3 Chemical reagents and equipment

Reagents: Purified water, anhydrous ethanol (purity >99.8%, Sigma-Aldrich, Germany); sodium carbonate, aluminum nitrate, sodium nitrite, sodium hydroxide, Folin-Ciocalteu reagent (analytical grade, Shanghai Yuanye Biotechnology Co., Ltd.), n-hexane, n-tridecane (HPLC grade, Sigma-Aldrich, Germany), C7-C40 n-alkane series (HJ894-2017, Anpel-Trace, China), anhydrous sodium sulfate (purity >98%, Shanghai Yisheng Biotechnology Co., Ltd.), dimethyl sulfoxide (analytical grade, Arabidopsis Biotechnology Co., Ltd., Chongqing), agar (Xiqiong Biotechnology, Beijing), glucose (purity >98%, Sichuan Baiochurui Biotechnology Co., Ltd., Chengdu), potatoes.

Instruments and Equipment: Balance (Suzhou Science Instrument Co., Ltd.); essential oil extractor, moisture analyzer (Sichuan Shubo Group Co., Ltd.), stereomicroscope (Leica Microsystems), grinder (XICHU Equipment Co., Ltd.), C18 preparative chromatography column [Agilent HC-C18 (4.6×250 mm, 10 μm)], gas chromatography-mass spectrometry (GC-MS) system (Agilent GC-MS 7890A-5975C), HP-5MS chromatography column (30 m×0.25 mm, 0.25 μm), laminar flow cabinet, constant temperature incubator, autoclave, induction cooker, microwave.

### 2.4 Extraction of essential oil

The oil content of fresh *Z. armatum* was determined according to the national standard GB/T 17527-2009, with the experiment repeated three times (ACFSMC, 2009). The oil content of dried *Z. armatum*: A certain amount of dried *Z. armatum* was ground using a grinder (XICHU Equipment Co., Ltd.) and passed through a 40-mesh sieve. Subsequently, 40 g of the resulting powder and 400 mL of pure water (1:10, v/v) were placed into a round-bottom flask of a volatile oil determination apparatus. The sample was heated with an electric heating mantle for distillation, which lasted for 3 hours at a rate of approximately 5 drops per minute. The distillate was collected in a graduated receiver tube, and anhydrous Na^2^SO^4^ was added to achieve water-oil separation. The volume of the oil was recorded, and the oil yield was calculated. The experiment was repeated three times (Qianqian et al., 2024). The extracted volatile oil was stored in amber glass bottles at -20°C, protected from light. The formula for calculating the essential oil extraction yield is as follows:


$$\text{Extractionrate of volatile oil }\left(\%\right)=\frac{\text{V}}{m}\times 100\%$$

V (mL) represents the volume of essential oil measured through hydrodistillation during the experiment, and m (g) represents the dry or wet weight of the sample used for extraction in the experiment.

### 2.5 Analysis of volatile components

GC-MS Analysis: Gas chromatography-mass spectrometry (GC-MS, Agilent GC-MS 7890A-5975C) was used to identify the components of the essential oil. The obtained *Z. armatum* essential oil was first dried with anhydrous sodium sulfate, then filtered through a 0.22 µm filter membrane and diluted 40 times with n-hexane before being injected. The internal standard used was n-tridecane (HPLC grade, Sigma-Aldrich, Germany). The GC-MS conditions were as follows: 1 mL of the sample solution was placed in an automatic injection vial, with an injection volume set to 10 µL. The chromatography column used was an HP-5MS (30 m×0.25 mm, 0.25 μm) elastic quartz capillary column (30 m×0.25 mm, 0.25 µm). The temperature program was as follows: column temperature set to 50°C (hold for 1 min), then ramped at 1°C/min to 75°C, held for 1 min, ramped at 6°C/min to 120°C, hold for 1 min, ramped at 1°C/min to 135°C, held for 1 min, then ramped at 15°C/min to 200°C and held for 5 min. Helium was used as the carrier gas, with a flow rate of 1.0 mL/min, and a split flow rate of 3 mL/min. The pressure was set to 7.6522 psi, and the injection port temperature was 250°C. The ion source used was electron impact (EI), with an ion source temperature of 230°C and a quadrupole temperature of 150°C (maximum 200°C), electron energy set at 70 eV, interface temperature at 280°C, and the mass scan range was 50–550 amu (Jingxuan et al., 2018). In this study, three methods were employed for the qualitative analysis of the components, including the calculation of retention index (RI) values, comparison with RI values reported in the literature, and the retrieval and matching of collected mass spectra against the NIST library to identify volatile components in the samples. Additionally, the content of each component was analyzed using the internal standard method.


$$\text{RI}=100\times \text{n}+\frac{{\text{log t}}_{\text{R}}\left(\text{x}\right)-{\text{log t}}_{\text{R}}\left(\text{n}\right)}{{\text{log t}}_{\text{R}}\left(\text{n}+1\right)-{\text{log t}}_{\text{R}}\left(\text{n}\right)}$$

tR(x) represents the retention time of the analyte, tR(n) is the retention time of the n-alkane with n carbon atoms, and tR(n+m) is the retention time of the n-alkane with (n+1) carbon atoms.

### 2.6 Establishment of fingerprint map

Fingerprint map technology refers to the chromatographic or spectroscopic maps obtained through appropriate processing of complex substances, followed by analysis using certain techniques (chromatography, spectroscopy). These maps reflect the chemical characteristics of the substances and are primarily used to evaluate the authenticity, quality, and stability of samples. Establishing a fingerprint map for the volatile components of *Z. armatum* is of significant importance for quality control, authenticity identification, and other aspects. The GC-MS mass spectra were analyzed using Agilent OpenLab CDS software. A fingerprint map was established for seven batches of *Z. armatum* samples using the “Traditional Chinese Medicine Chromatographic Fingerprint Similarity Evaluation System (2012 edition).” A reference map was generated using the median method, with a time window width of 0.1. After multi-point calibration and automatic matching, the volatile component fingerprint map of *Z. armatum* was obtained.

### 2.7 The attraction-avoidance experiment of *Z. armatum* essential oil on *T. castaneum*

The attraction-avoidance experiment on *T. castaneum* (male and female) was conducted using the *Z. armatum* essential oil obtained from previous experiments. The experiment followed the method described by Jilani et al (Jilani and Saxena, 1990), with some modifications. The specific procedure is as follows: First, preliminary experiments were conducted using four solvents: DMSO, acetone, methanol, and ethanol, to determine ethanol as the solvent for the essential oil. Then, several 4–7 day-old adult beetles were selected and kept in a wheat flour incubator for 24 hours. Filter paper (9 cm in diameter) was placed at the bottom of a 9 cm diameter petri dish, and the paper was divided into two halves, completely covering the bottom of the dish with a clear cutting line in the middle.

The *Z. armatum* essential oil was diluted with 75% ethanol to prepare solutions with volume fractions of 1%, 3%, and 5%. Using a pipette, 300 μL of the *Z. armatum* essential oil solution was evenly spread on the filter paper on the right side of the petri dish, and left to stand for thirty minutes to allow the solvent to evaporate. Ten healthy male and ten healthy female *T. castaneum* adults (7 days post-eclosion) were selected and placed in a petri dish. The chosen beetles displayed robust body condition, high mobility, and quick responses to stimuli (e.g., light and vibration). The petri dish was then placed in an incubator at room temperature and away from light. Every 12 hours, the number of *T. castaneum* beetles on the left and right sides of the petri dish was recorded, and the avoidance rate was calculated. The negative control was an ethanol solution with the same volume. All experiments were repeated three times. The avoidance rate was calculated using the following formula:


$$\text{Avoidance rate}\;(\%)=\frac{\text{n}1-\text{n}2}{\text{n}1}\times 100\%$$

Where n1 is the number of insects placed in the petri dish, and n2 is the number of insects observed on the filter paper coated with the essential oil solution.

### 2.8 The antifungal experiment of *Z. armatum* essential oil against *C. cassiicola*

The growth inhibition experiment on *C. cassiicola* was conducted using the *Z. armatum* essential oil obtained in previous experiments. The *Z. armatum* essential oil was diluted with a 2.5% dimethyl sulfoxide (DMSO, analytical grade, Arabidopsis Biotechnology Co., Ltd., Chongqing) solution to concentrations of 0.5%, 0.1%, and 0.05%. Using a pipette, 5 mL of the solution was added to 25 mL of PDA medium, thoroughly mixed to ensure uniformity. The 30 mL mixture was evenly poured into three Petri dishes. After the medium solidified, the *C. cassiicola* strain, obtained through isolation and purification, was inoculated. The colony diameter was observed and recorded every 24 hours (Na et al., 2017). The negative control was a 2.5% DMSO solution, and the blank control was distilled water.

### 2.9 Data processing

The data were analyzed using one-way ANOVA in SPSS 22 software. The volatile component fingerprint of *Z. armatum* was established using the “Traditional Chinese Medicine Chromatographic Fingerprint Similarity Evaluation System (2012 version)”. Principal component analysis (PCA) was performed based on the content of each component in the essential oil. Correlation heatmaps were generated using the OmicShare tool (https://www.omicshare.com/), and all other figures were created with Origin Pro 2022.

## 3 Results and analysis

### 3.1 Morphological changes in the fruits of *Z. armatum* at different harvesting periods

**Figure 2:**
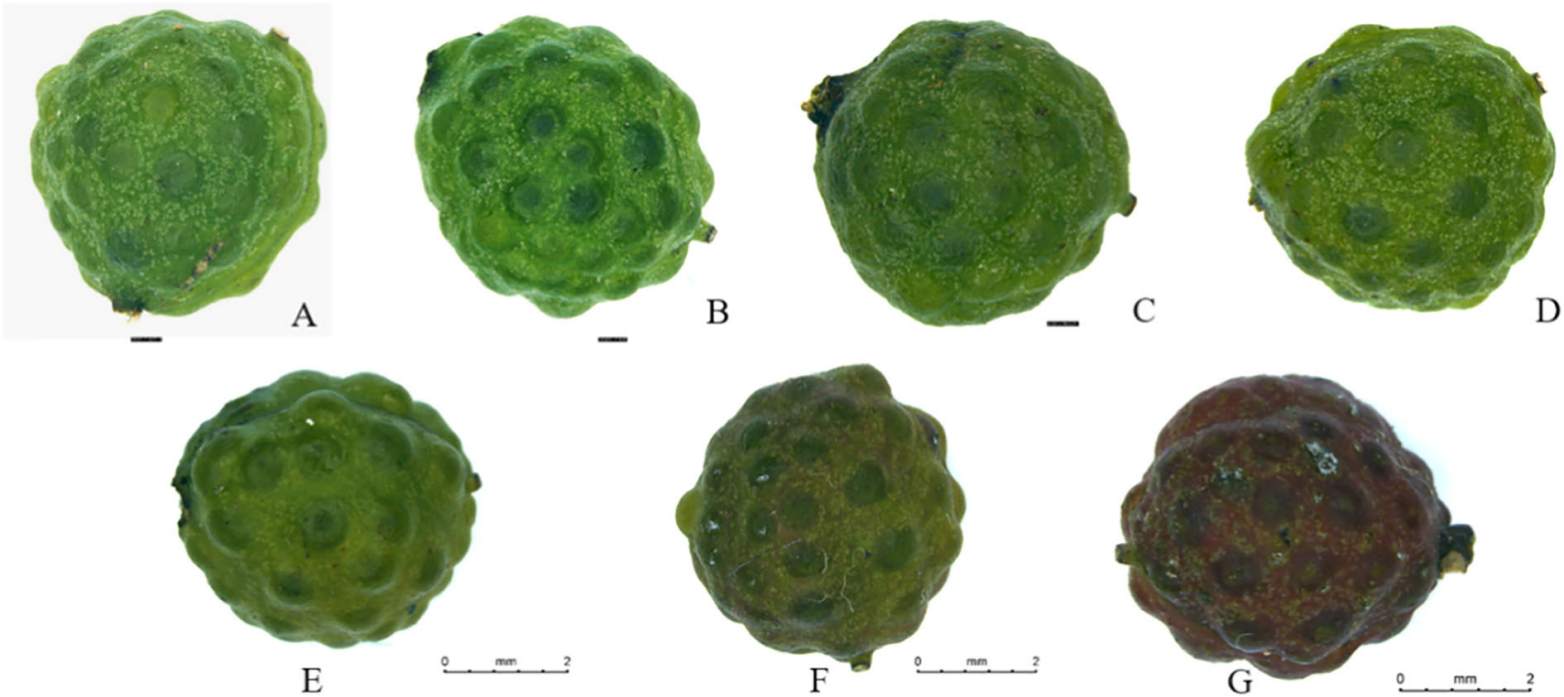
Morphology of *Z. armatum* fruits at different harvest periods [**(A-G)**, t1-t7 periods respectively].

Photographs of *Z. armatum* fruits at different harvesting periods were taken under a stereomicroscope, clearly revealing the morphological changes in the fruits (as shown in **Figure 2**). In the early stages, the fruits exhibit a bright green exterior with slightly raised oil glands. As the growth and development process progresses, the color gradually deepens, and the peel turns yellowish-green with more prominent oil glands. By the t7 period, the fruits have transitioned from yellowish-green to red, displaying a more oily sheen on the surface, indicating that the fruits are fully mature at this stage.

### 3.2 Analysis of volatile components

The volatile components in *Z. armatum* essential oil were analyzed by GC-MS. For statistical analysis, we selected volatile compounds meeting the following criteria: (1) concentration ≥2 mg/mL and (2) match confidence >95% against the NIST database. Through this approach, we initially identified 26 volatile constituents. The compounds are arranged in order of their elution sequence. As shown in **Table 1**, the relative content of various components varied across different growth stages. Linalool (399.92–529.48 mg/mL), D-Limonene (126.94–200.14 mg/mL), and Sabinene (71.26–140.64 mg/mL) were the three most abundant volatile components in the essential oil of *Z. armatum* pericarp, followed by β-Myrcene (16.42–29.67 mg/mL), Nerolidol (3.18–15.07 mg/mL), and Terpinen-4-ol (6.59–13.24 mg/mL). These volatile compounds are the primary components of *Z. armatum* essential oil and contribute to its rich herbal, piney, and fresh aroma (Xiya et al., 2022). Among the three main volatile components, the relative content variations of Sabinene and D-Limonene closely mirrored the overall changes in the essential oil yield. Sabinene reached its minimum relative content of 71.26 mg/mL at the t5 stage and its maximum of 140.64 mg/mL at the t7 (September 28) stage. Similarly, D-Limonene showed a minimum relative content of 126.94 mg/mL at the t5 stage and a maximum of 200.14 mg/mL at the t7 stage, though there was no significant difference compared to the t2 stage. In contrast, Linalool had the lowest relative content of 399.92 mg/mL at the t1 stage and the highest of 529.48 mg/mL at the t5 stage.

**Table 1:** Volatile content at different harvest times.

Compound	RI^a^	CAS	Compound content (mg/ml)							Significance level
			t1	t2	t3	t4	t5	t6	t7	P-value
α-Pinene	917	80-56-8	8.56636288	9.68013504	5.08812672	6.45506632	4.09436612	8.06151152	10.72558572	*P<0.01*
Sabinene	970	3387-41-5	119.563481	125.9186286	77.48707836	92.6637054	71.2609966	117.2219146	140.6438818	*P<0.01*
β-Myrcene	994	123-35-3	26.31376088	28.77508788	18.47107412	21.18433656	16.41665564	26.36525388	29.67266176	*0.22*
α-Phellandrene	1004	99-83-2	4.72410976	4.57629132	3.47297924	3.27007232	2.37972444	4.628221	4.61221724	*P<0.01*
D-Limonene	1020	5989-27-5	185.8556566	198.325901	147.9515164	159.6444335	126.9446598	188.1898186	200.1371184	*P<0.01*
β-Ocimene	1034	13877-91-3	9.11552192	9.24845536	6.75645952	7.055808	5.5082226	9.67103252	9.68026292	*P<0.01*
γ-Terpinene	1040	99-85-4	9.227553	9.32074512	6.48201852	7.12339924	6.34593132	9.52492348	10.25516676	*P<0.01*
Terpinolene	1064	586-62-9	4.09290364	4.74538744	2.13091712	2.79945372	2.54815428	3.78787432	4.67278668	*0.106*
Linalool	1087	78-70-6	399.9164031	401.1676404	451.2484544	471.042912	529.4837364	409.1016716	414.3012924	*P<0.01*
Terpinen-4-ol	1170	562-74-3	12.47652984	8.72399972	6.5909376	7.10201308	10.91187824	12.69601744	13.243583	*P<0.01*
α-Terpineol	1187	98-55-5	4.94793692	-	-	-	3.87853496	8.31836416	-	*P<0.01*
2-Penten-4-yn-1-ol	824	5557-88-0	0.12338144	-	-	-	-	-	-	*P<0.01*
Linalyl acetate	1290	115-95-7	8.853537	7.71461584	5.12503416	4.950635	6.58939408	8.74490676	10.2155162	*0.022*
Isobornyl acetate	1280	125-12-2	0.29128196	-	-	-	-	0.5852336	-	*P<0.01*
Nerylacetate	1361	141-12-8	0.15263684	-	-	-	-	-	0.40700808	*P<0.01*
β-elemene	1390	515-13-9	2.30736004	1.51454716	2.62379924	2.12108488	2.25563448	2.258364	1.947868	*0.05*
Caryophyllene	1411	17627-40-6	4.71252376	3.72933348	6.96463416	5.82680692	6.144992	5.2360978	4.61680972	*P<0.01*
Humulene	1434	6753-98-6	2.86468724	2.88202008	4.18502872	3.49848908	3.88182936	3.39041792	2.76497592	*P<0.01*
Germacrene D	1472	23986-74-5	7.37283916	4.70815148	11.9498594	9.1824182	10.28684032	8.48448824	6.78569596	*P<0.01*
germacrene B	1417	15423-57-1	3.38432372	-	-	-	-	-	3.10289604	*P<0.01*
Nerolidol	1557	7212-44-4	3.4929832	4.22766696	15.0714184	3.95416096	11.15071252	8.43784564	3.1782044	*P<0.01*
Geranyl phenylacetate	1432	102-22-7	0.0381592	-	-	-	-	-	0.37216232	*P<0.01*
α-thujene	940	2867-05-2	2.22437176	4.55050472	-	3.4911792	2.290896	-	-	*P<0.01*
α-Terpinene	1012	99-86-5	3.55909864	3.65208396	2.91572732	3.28140164	2.90115724	3.4890124	4.13389516	*P<0.01*
2-Carene	1031	554-61-0	4.12228368	1.83899256	-	-	-	5.0391162	-	*P<0.01*
bicyclogermacrene	1482	24703-35-3	-	-	5.43166316	4.22497996	4.58936464	-	-	*P<0.01*

Other volatile components with lower concentrations generally show a pattern similar to D-Limonene and Sabinene, decreasing and then increasing as the developmental stages progress, exhibiting a “concave” shape. These include α-Pinene, β-Myrcene, Terpinen-4-ol, Linalyl acetate, etc. Some volatile components, such as α-Phellandrene, β-Ocimene, and γ-Terpinene, show little variation throughout the entire growth period. Overall, the content of volatile oil and the main aromatic compounds in the fruit peel of *Z. armatum* accumulate most abundantly in August, making it the optimal time for harvesting for further processing and utilization.

### 3.3 Establishment of the volatile component fingerprint

The “Chinese Medicine Chromatographic Fingerprint Characteristic Map Similarity Evaluation System (2012 version)” was used to establish fingerprint maps for *Z. armatum* at seven different maturity stages. The shared fingerprint map for the seven stages was matched, and the relative peak areas of the shared fingerprint map were analyzed.

The similarity between the samples from different periods is relatively low between t5 and t2, and between t5 and t7, with similarities of 0.966 and 0.964, respectively (sample similarity comparisons are shown in **Table 2**). This indicates that the volatile components of t5 differ slightly from those of the other two periods. Overall, the similarity between the seven samples is above 0.96, suggesting that the volatile components of *Z. armatum* from different periods exhibit good consistency.

**Table 2:** Comparison of similarity of *Z. armatum* fingerprints at different maturity stages.

Similarity	t1	t2	t3	t4	t5	t6	t7	Reference fingerprint map
t1	1	0.999	0.99	0.993	0.975	1	0.999	0.998
t2	0.999	1	0.984	0.988	0.966	0.999	1	0.995
t3	0.99	0.984	1	1	0.996	0.989	0.982	0.997
t4	0.993	0.988	1	1	0.994	0.992	0.987	0.998
t5	0.975	0.966	0.996	0.994	1	0.973	0.964	0.987
t6	1	0.999	0.989	0.992	0.973	1	0.999	0.998
t7	0.999	1	0.982	0.987	0.964	0.999	1	0.994
Reference fingerprint map	0.998	0.995	0.997	0.998	0.987	0.998	0.994	1

**Figure 3:**
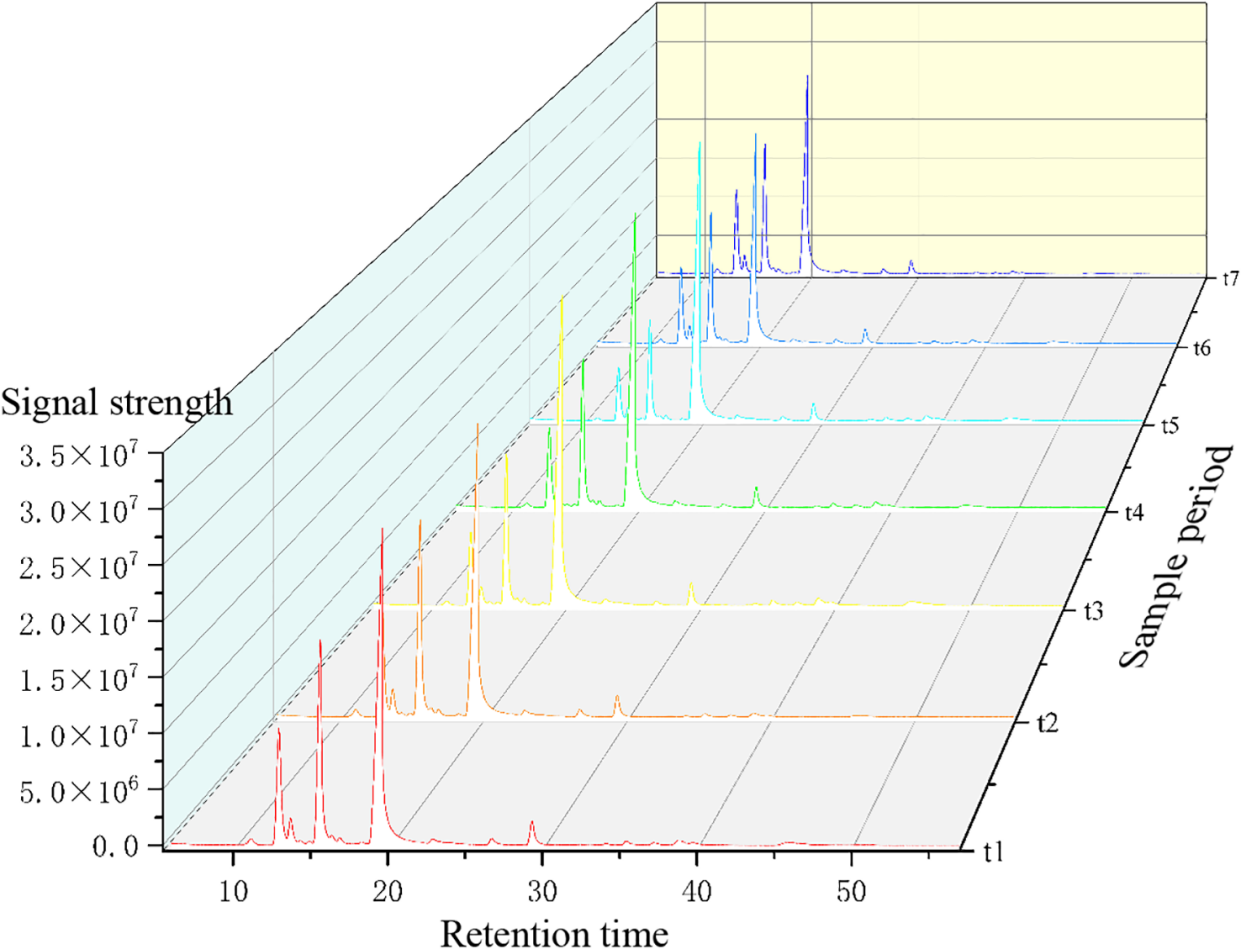
Fingerprint of *Z. armatum* volatile components.

**Figure 4:**
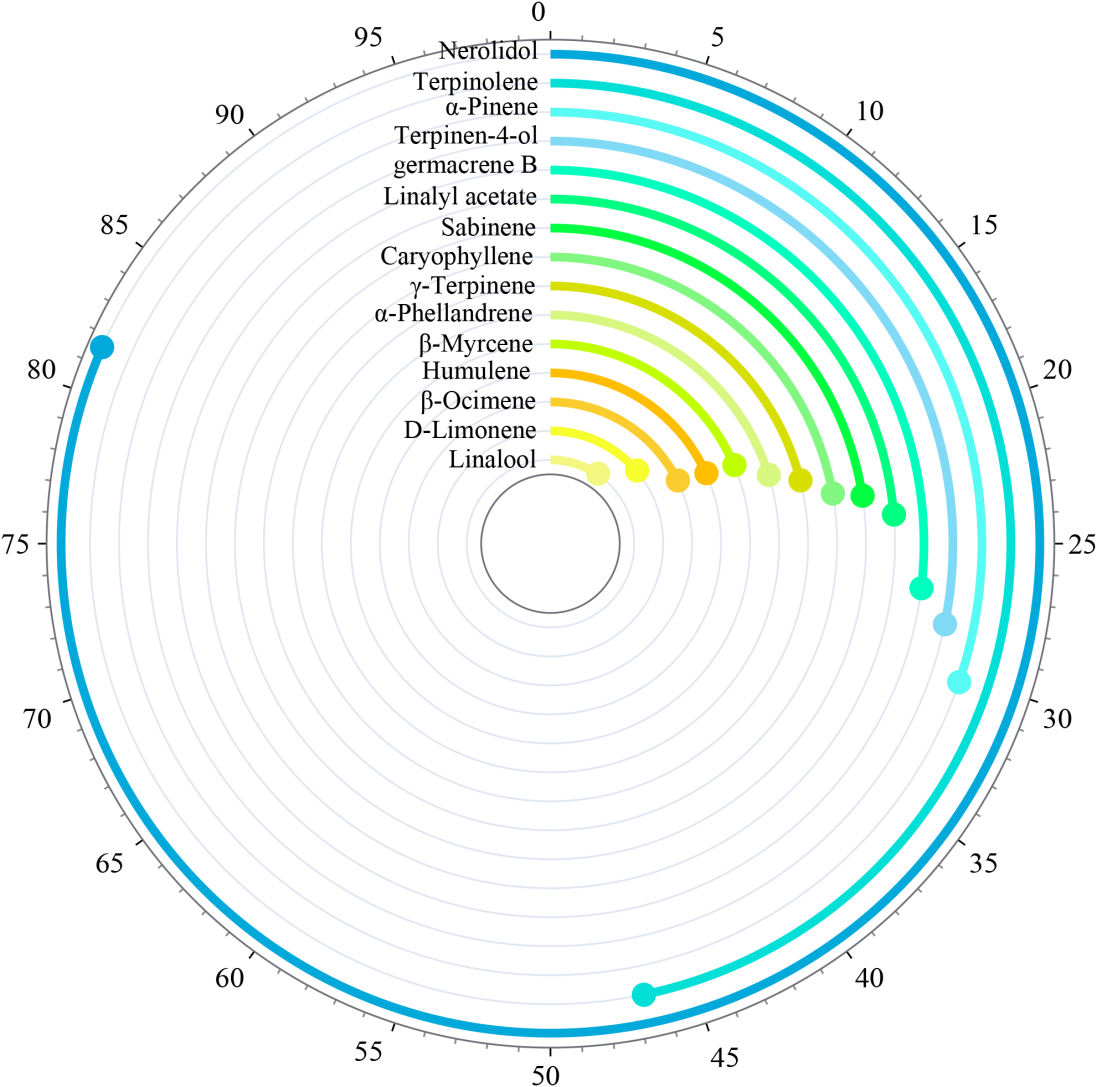
Relative peak area of fingerprint of volatile components.

The fingerprint spectrum is shown in **Figure 3**. The relative peak area of the fingerprint spectrum represents the variation of target substances from t1 to t7. By comparing with the mass spectrum, 15 common mass spectrum peaks with relative peak areas were identified. As shown in **Figure 4**, the relative peak area of Linalool is 11.79%, indicating that its variation is the smallest during the fruit’s growth and development process. D-Limonene and β-Ocimene also show relatively small changes. In contrast, the relative peak area of Nerolidol is 81.97%, showing the largest variation during the fruit’s growth and development. Following Nerolidol is Terpinolene. The significant changes in these two components, which have strong citrus aromas, may be one of the reasons for the large flavor differences observed in *Z. armatum* at different maturity stages.

### 3.4 Principal component analysis

**Figure 5:**
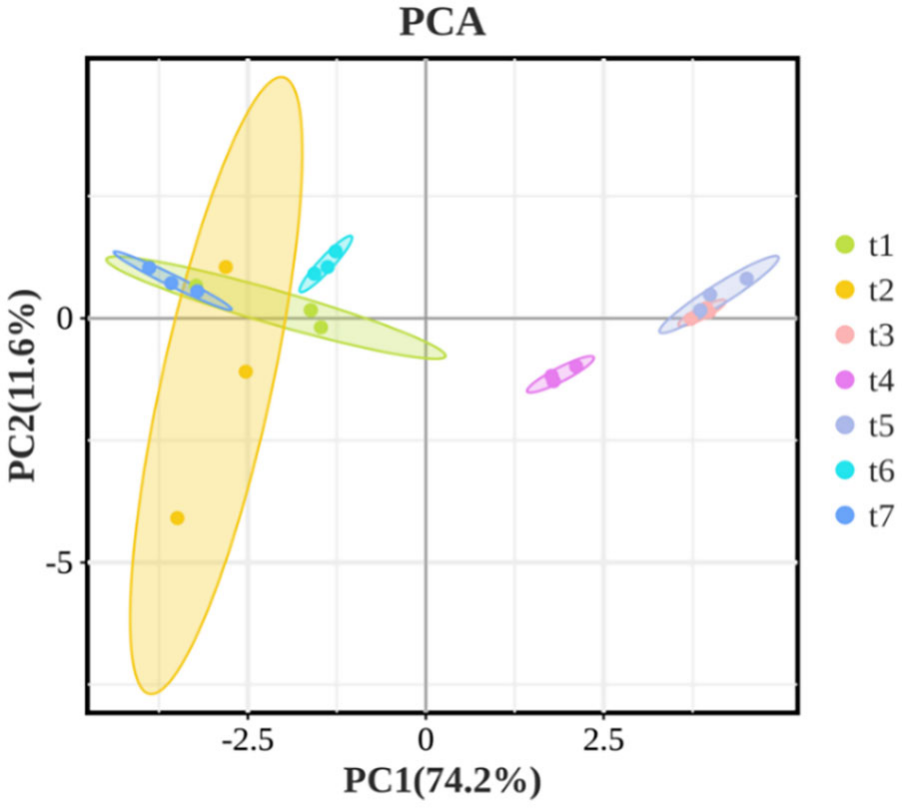
PCA based on volatile components.

To preliminarily understand the differences and similarities of volatile compounds in *Z. armatum* at different harvest stages, the PCA analysis of the volatile components is shown in **Figure 5**. The first principal component (PC1) explains 74.2% of the variance, while the second principal component (PC2) explains 11.6%. In the **Figures 5**, samples t1, t2, t6, and t7 cluster together and are distributed on the negative half of PC1, while samples t3, t4, and t5 cluster on the positive half of PC1. This suggests that the volatile oil components of *Z. armatum* during t1 (May 26), t2 (June 16), t6 (September 9), and t7 (September 28) are similar, with significant differences compared to those at t3 (July 3), t4 (July 18), and t5 (August 4). This indicates that the volatile components of the pepper peel continuously change in the early stages of maturity and significantly differ from other growth stages. As the fruit matures, the accumulation of volatile components gradually slows down and tends to stabilize.

### 3.5 Analysis of insect repellent experiment results

**Figure 6:**
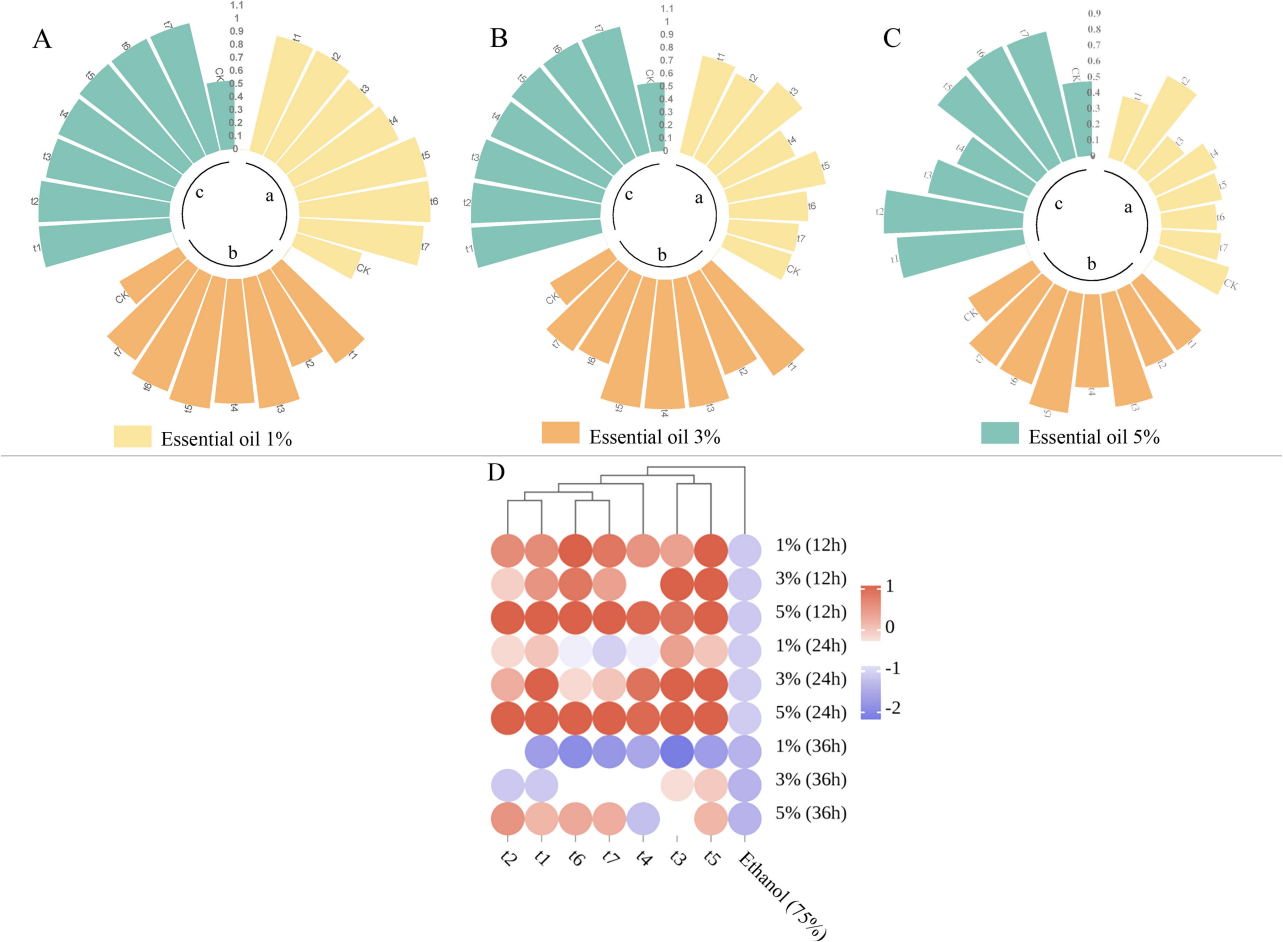
Repellent rates and clustering heatmap of *Z. armatum* essential oil at different concentrations against *T. castaneum* after 12, 24, and 36 hours of application. [**(A)** Repellent rates at 12 hours; **(B)** Repellent rates at 24 hours; **(C)** Repellent rates at 36 hours; **(D)** Clustering heatmap].

The results of the repellent experiment with *Z. armatum* EO against *T. castaneum* are presented in **Figure 6** and **Table 3**. As the treatment time increased, the repellent rate of the essential oil at different concentrations significantly decreased. The highest repellent rate of 100% at 12 hours post-treatment dropped to a maximum of 88% at 36 hours post-treatment, with a significant difference compared to the control group. At 36 hours post-treatment, the 5% concentration of *Z. armatum* EO still maintained a repellent rate of nearly 80% against *T. castaneum*. The 3% concentration of *Z. armatum* EO showed relatively high repellent activity (65%-100%) at 12 hours and 24 hours post-treatment, but the activity was lost after 36 hours. The 1% concentration of *Z. armatum* EO showed significant repellent effects against *T. castaneum* only within the first 12 hours post-treatment.

**Table 3:** The repellent rate of different concentrations of *Z. armatum* essential oil to Tribolium castaneum after 12, 24 and 36 hours of application.

Time	Pharmaceuticals	Concentration	Sample sampling period						
			t1	t2	t3	t4	t5	t6	t7
12h	Essential oil	1%	0.9	0.9	0.85	0.88	1	1	0.95
		3%	0.88	0.75	1	0.95	1	0.95	0.85
		5%	1	1	0.97	0.98	1	1	1
	Ethanol (CK)	60%	0.52						
24h	Essential oil	1%	0.77	0.73	0.85	0.61	0.77	0.61	0.54
		3%	1	0.82	1	1	1	0.73	0.77
		5%	1	1	1	0.98	1	1	1
	Ethanol (CK)	60%	0.53						
36h	Essential oil	1%	0.40	0.62	0.30	0.43	0.40	0.35	0.38
		3%	0.52	0.52	0.72	0.59	0.76	0.64	0.64
		5%	0.80	0.88	0.62	0.50	0.80	0.83	0.82
	Ethanol (CK)	60%	0.47							

(The data is the average of three experiments).

The EO repellent experiment data were normalized and a clustering heatmap was generated, as shown in **Figure 6**. The *Z. armatum* EOs from different maturation stages were grouped into three categories: t1 (May 26), t2 (June 16), t6 (September 9) and t7 (September 28)were clustered into one group; t3 (July 3) and t5 (August 4), into another group; and t4 (July 18) was grouped separately. The clustering results are consistent with the previous analysis of volatile components. This indicates that the primary repellent mechanism of the essential oil relies on fumigant toxicity rather than contact toxicity, providing a reference for the development of insect repellent formulations based on *Z. armatum* essential oil (**Table 4**).

**Table 4:** Main effects and interaction effects results.

Factors	F-value	p-value	Significant effect (α=0.05)	Effect Size (η²)
Time	45.72	<0.001	Significant	0.38
Concentration	28.15	<0.001	Significant	0.25
Period	5.64	<0.001	Significant	0.12
Time × Concentration	4.33	0.002	Significant	0.09
Time × Period	1.98	0.061	Not significant	-
Concentration × Period	1.12	0.341	Not significant	-
The third-order interaction	0.87	0.512	Not significant	-

### 3.6 Analysis of antifungal experiment results

**Figure 7:**
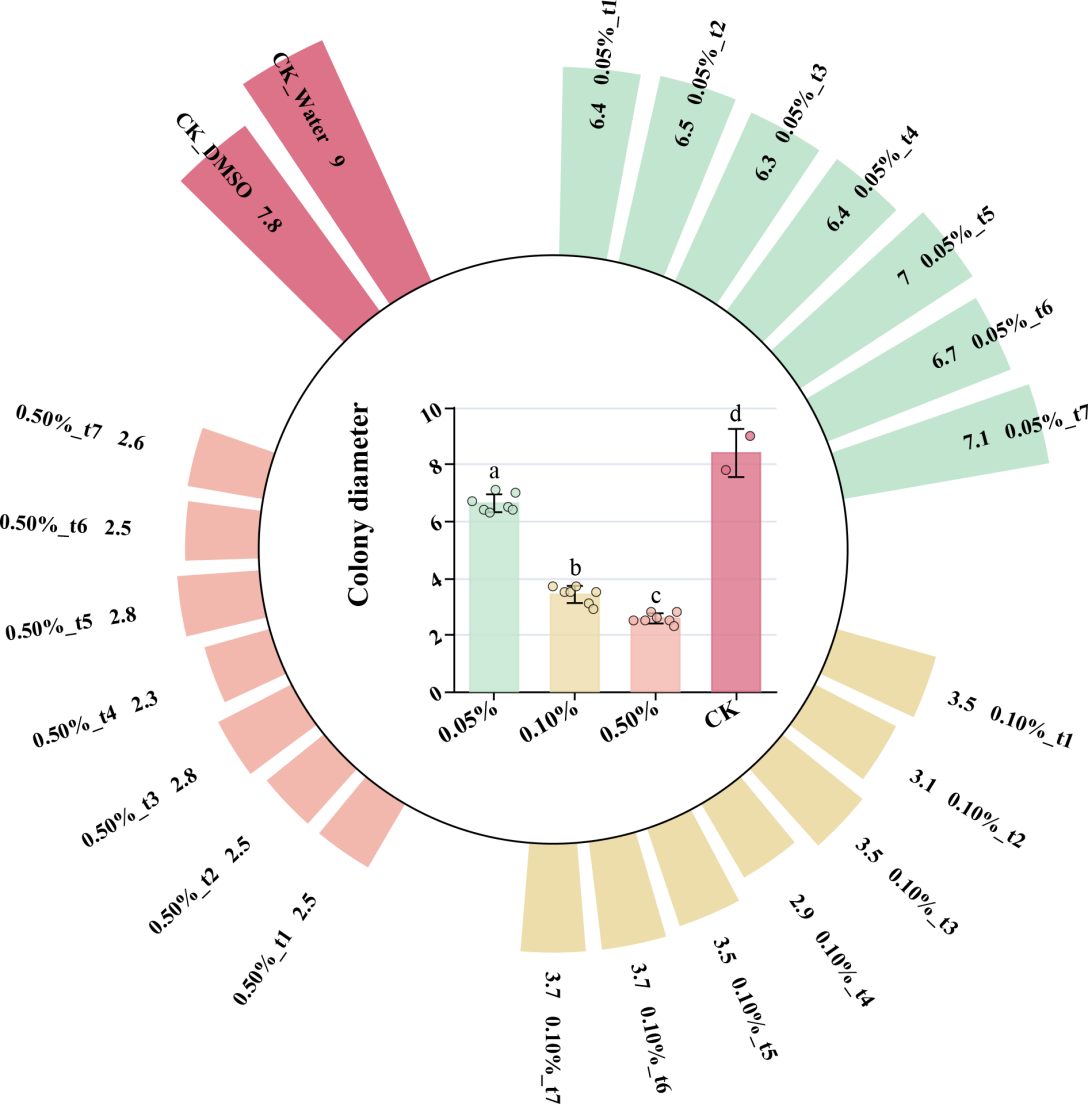
After three days of culture, the colony diameter of *C. cassiicola* was affected by different concentrations of *Z. armatum* essential oil solution.

The essential oil of *Z. armatum* from different maturity stages exhibited significant inhibitory effects on *C. cassiicola*, with the inhibitory effect varying significantly at different concentrations of the essential oil solution. The 0.5% concentration showed the strongest antifungal effect. After 3 days of culture, the colony diameter in the negative control group was 64.1%-70.51% larger compared to the 0.5% group, 55.13%-62.82% larger compared to the 0.1% group, and 8.97%-19.23% larger compared to the 0.05% group (**Figure 7**). The increase in essential oil concentration significantly enhanced the antifungal effect against *C. cassiicola*. At 0.5% and 0.1% concentrations, only the essential oil from the t4 stage showed significant differences in antifungal effect compared to the other stages.

**Figure 8:**
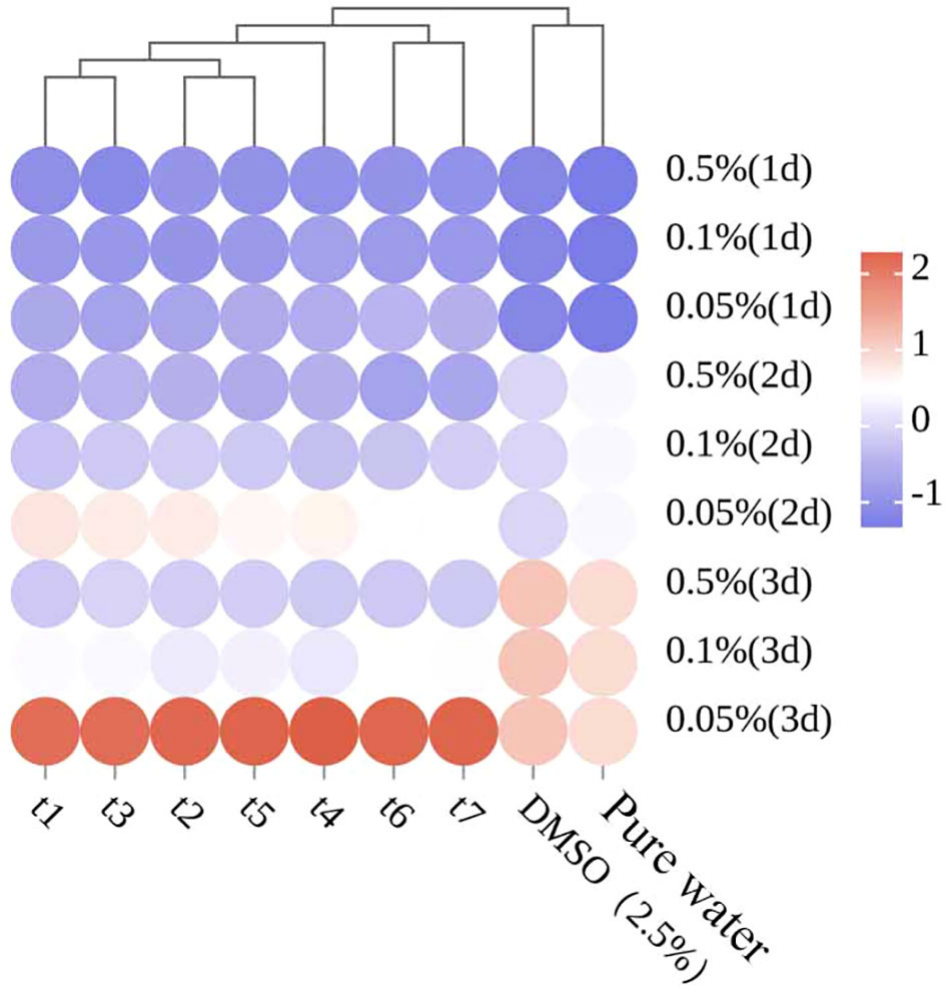
Cluster thermogram analysis of bacteriostatic experiment.

After normalizing the antifungal data of the EOs, a cluster analysis was performed, and the results are shown in **Figure 8**. The samples from t1 (May 26), t2 (June 16), t3 (July 3), and t5 (August 4) were grouped together, while t4 (July 18) was grouped separately, and t6 (September 9) and t7 (September 28) were grouped together. These results differ from the previous clustering analysis, further indicating that the antifungal properties of the essential oil are not solely influenced by the volatile components or any single component, but rather by the combined effect of multiple components.

## 4 Discussion

The production, composition, and content of secondary metabolites in plants are influenced by various conditions. Based on market data of volatile oil compounds from Chinese *Z. armatum*, the projected demand for 2025–2030 is estimated at 40,000-47,500 thousand metric tons, with China’s market share accounting for 15%-18% of the global market. The primary growth drivers stem from expanding applications in: Food and Flavoring Industry, Cosmetics and Personal Care, Pharmaceuticals and Traditional Chinese Medicine, Green Pesticides and so on (Research Report on Monitoring and Analysis of Chinese Zanthoxylum Essential Oil Market (2025 -2030), 2025). For economically important plants with commercial value, optimizing planting time and harvest periods can help obtain commercial products that better meet market demands (A. Cristina et al., 2008). The volatile components of *Z. armatum* are primarily stored in the fruit peel, with its linalool and d-limonene content being influenced by precipitation-related climatic factors (Qianqian et al., 2024). Therefore, we analyzed the volatile oil components and their content in the fruit peel of *Z. armatum* from different periods in Nanchong, Sichuan Province. This study aimed to explore the accumulation pattern of volatile oil substances in *Z. armatum* at different stages of growth and development by measuring the volatile components and their content. We conducted GC-MS analysis of the volatile components in *Z. armatum* essential oil using an internal standard method, preliminarily identifying and quantifying 28 volatile constituents in relative content. However, since reference standards were not employed for verification, the experimental results have certain limitations. The results show that the accumulation of volatile components occurs before the t5 stage. Studies have shown that in the early developmental stages, the stimulating and aromatic substances produced by *Zanthoxylum bungeanum* are mainly stored in the leaves and gradually transfer to the fruit peel in the later developmental stages (Lei et al., 2019). Therefore, the volatile components in the fruit may accumulate in the later stages of development. *Z. armatum* harvested in July and August has a higher content of volatile components compared to other periods, making it the preferred choice for processing and making other products. The principal component analysis results show that there are significant differences in the volatile components of the pepper fruits between the t3 (July 3), t4 (July 18), t5 (August 4) stages and the t1 (May 26), t2 (June 16), t6 (September 9), t7 (September 28) stages. In the early stages of fruit maturation, plants exhibit vigorous metabolic activity, producing a significant amount of monoterpenes (Telci et al., 2009) such as α-Pinene, Sabinene, β-Myrcene, α-Phellandrene, and D-Limonene. As the fruits of *Z. armatum* mature, the content of more structurally complex compounds, such as alcohols (Kamel et al., 2007) and esters (Moghaddam et al., 2015), increases. For instance, compounds like Linalyl acetate, Terpinen-4-ol, and α-Terpineol are relatively abundant during the fruit’s maturation period. The significantly lower oil yield from dried *Z. armatum* pericarps during the mid-maturity stage may also be related to the physiological metabolism of the plant. In the early stage, low-molecular-weight volatile compounds accumulate rapidly. During the mid-stage, high-molecular-weight lipid compounds are generated. By the fully mature stage, the content of volatile alcohols and esters in the fruits increases.

*Z. armatum* EO has a strong repellent effect on *T. castaneum*, and the effect changes in a gradient with concentration, indicating that the repellent effect is positively correlated with the essential oil content to some extent. Low concentrations of *Z. armatum* EO solution can also produce a strong repellent effect on *T. castaneum* at the early stage of application. The volatile compounds in the EO, such as monoterpenes, usually have a strong odor, which may stimulate *T. castaneum*, causing it to exhibit avoidance behavior. Furthermore, because the odor threshold of various volatile components is relatively low, even at low concentrations, these compounds can induce a repellent response in *T. castaneum* (Daokui, 2023). Studies have shown that substances like α-pinene and laurene can induce a strong repellent effect on *T. castaneum* (Kim et al., 2010; Maurya et al., 2018). This all demonstrates that the volatile components of *Z. armatum* EO have a good repellent effect on *T. castaneum*. However, the effect decreases as the active ingredients volatilize. Preventing the evaporation of the essential oil could significantly extend its repellent effectiveness. Unlike previous studies, no mortality of *T. castaneum* adults was observed in this experiment. In previous experiments, *Z. armatum* essential oil solutions applied to the abdomen of *T. castaneum* also did not result in insect death within 48 hours. This suggests that the low content of Terpinen-4-ol and the absence of decanal, a compound with strong insecticidal activity, may be the reasons for the lack of mortality in the tested insects (Yang et al., 2019). Another possible reason is that *T. castaneum* perceives chemical signals in the surrounding environment through sensory organs such as its antennae (Feng et al., 2014). The volatile chemicals in the EO may only interact with the receptors on the insect’s sensory organs (Thomas S. et al., 2012), triggering a repellent response, but not enough to cause death. The specific activity and mechanism need to be determined through more detailed experiments and studies.

In the antimicrobial experiment, the baseline antifungal activity of *Z. armatum* EOs was determined by comparing the antifungal activities of EOs extracted at different sampling periods and against the negative control. However, since no positive control was included, the practical application value of the EOs could not be fully validated. The results showed that *Z. armatum* EOs from different maturity stages exhibited significant inhibitory effects on *C. cassiicola*, and the antimicrobial activity increased with higher EO concentrations. A study by Nirmala et al. showed that methanol extracts of the peel, seeds, and bark of *Z. armatum* also exhibited antifungal activity against microorganisms such as *Bacillus subtilis*, *Enterococcus faecalis*, *Staphylococcus aureus*, and *Staphylococcus epidermidis* (Phuyal et al., 2020). Since the best antifungal effect was observed during the t4 period, the EO extracted from *Z. armatum* harvested in mid-July showed more prominent antifungal activity. Previous studies have shown that the antifungal effect is positively correlated with the content of linalool (Wei et al., 2023). In this study, the t4 period, which exhibited the best antifungal effect, was not the period with the highest linalool content. However, by comparing samples from other periods, it was found that although the relative contents of various compounds in the t4 period were not the highest, they still ranked among the top. Examples include Linalool, Germacrene D, Nerolidol, (+)-Citronellal, and Bicyclogermacrene. These compounds exhibit varying degrees of antibacterial and antifungal activity (M J. et al., 2009; Ricardo et al., 2011; Xiuming et al., 2023). We hypothesize that the specific compositional ratio of components at t4 may lead to synergistic enhancement (e.g., through multi-target disruption of cell membranes), whereas the isolated increase in linalool at t5, accompanied by a decline in other constituents, could result in reduced overall bioactivity. However, this conclusion requires further experimental validation. Studies on the essential oil of *Tetraclinis articulata* have shown that the combination of antibiotics with extracts from this species is more effective, exhibiting 100% synergistic or partial synergistic effects against the tested bacteria (Djouahri et al., 2014). Therefore, the antifungal effects of different chemical components are inconsistent. Investigating the synergistic and antagonistic interactions between different chemical components in terms of antifungal activity can serve as a new direction for future research.

In the insect repellent and antifungal experiments, different concentrations of *Z. armatum* essential oil exhibited insecticidal/antifungal activity against *T. castaneum* and *C. cassiicola*, with a gradient relationship as the concentration increased. *Z. armatum* essential oil is characterized by high levels of oxygenated monoterpenes and monoterpene hydrocarbons. These monoterpenes are common components of essential oils and are used as fragrances and flavorings in cosmetics, perfumes, pharmaceuticals, and food products (Domenico et al., 2005). The EO used in the *in vitro* antifungal tests contains a significant number of monoterpenes. The main components in *Z. armatum* EO, such as Linalool, D-Limonene, and Sabinene, have been proven to exhibit strong insecticidal/antifungal properties (Djenane, 2015; Xue et al., 2020). This may be the basis for the strong insecticidal and antifungal activities of *Z. armatum* EO. Regarding the mechanism of action of the EO, it may involve interactions between enzymes and membrane proteins. In some cases, the active compounds in the EO can penetrate the lipid bilayer of the cell membrane, increasing the cell’s permeability and leading to the leakage of important cellular contents (Alvarez-Martinez et al., 2021; Maurya et al., 2022). The antimicrobial activity data herein provide a foundation for future mechanistic studies, though specific targets (e.g., membrane disruption/enzyme inhibition) require validation via component-specific assays. *Z. armatum* EO, rich in these components, can become a key part of natural plant-based insecticidal and antifungal agents. Additionally, the *Z. armatum* EO from the t4 period shows superior antifungal effects. For the development of antifungal drugs based on *Z. armatum* EO, it is recommended to harvest the fruits in mid-July.

## 5 Conclusion

The different growth and development stages are important factors influencing the accumulation of secondary metabolites in *Z. armatum* fruits. This study performed GC-MS analysis on the *Z. armatum* EOs from Nanchong City, Sichuan Province, and conducted insect-repellent and antifungal experiments using EOs from different stages. The GC-MS results showed that the accumulation of volatile compounds occurred before the t5 stage, as low-molecular-weight monoterpenes were rapidly generated in the early fruit ripening stages, while alcohols and esters accumulated in later stages. The EO content and its major fragrance components accumulated richly in July and August. Both insect-repellent and antimicrobial experimental results indicated that the EO from the t4 stage had higher insect-repellent and antifungal activity. Therefore, the insect-repellent and antimicrobial effects of the EOs may result from the synergistic or antagonistic interactions among various compounds. This study provides a reference for utilizing *Z. armatum*’s different commercial values based on harvest periods and offers a research direction for using plant EOs as natural insect-repellent/antimicrobial agents. Future research can delve into analyzing the interaction mechanisms between different compounds in the essential oil and the underlying mechanisms of its insect-repellent and antifungal activities.

## Data Availability

The datasets presented in this study can be found in online repositories. The names of the repository/repositories and accession number(s) can be found in the article/supplementary material.
